# The role of tonifying kidney decoction and acupuncture in the treatment of Alzheimer’s disease: A network meta-analysis

**DOI:** 10.1097/MD.0000000000031243

**Published:** 2022-11-18

**Authors:** Xin-Chen Wang, Chen-Liang Chu, Kuan Lu, Xi Chen, Xiao-Qian Jin, Shi-Jian Quan

**Affiliations:** a Department of Pharmaceutical Engineering, College of Food and Pharmaceutical Engineering, Zhaoqing University, Zhaoqing, Guangdong Province, China; b Department of Epidemiology and Statistics, School of Public Health, Medical College, Zhejiang University. Hangzhou, Zhejiang Province, China; c Rehabilitation Medicine Department, Zhuhai Hospital Affiliated with Jinan University, Xiangzhou District, Zhuhai City, China; d School of Pharmaceutical Sciences, Guangzhou University of Chinese Medicine, Panyu District, Guangzhou City, China.

**Keywords:** acupuncture, Alzheimer’s disease, Chinese herbal compound, network meta-analysis, tonifying kidney decoction

## Abstract

As one of the chronic neurological degenerative diseases with the highest incidence of amnesia and dementia, Alzheimer’s disease (AD) carried out the clinical treatment based on the 2 traditional Chinese medicine (TCM) of Chinese herbal compound and acupuncture (AP). With the vigorous development of TCM, doctors are facing the problem of choosing TCM or western medicine in clinical work. Hence there is an urge to make pairwise comparisons among these interventions to provide evidence for clinical practice.

**Objective:**

The used efficacy of the 2 TCM methods and combined with donepeziline were compared to compile the best treatment through network meta-analysis.

**Methods:**

Patients diagnosed with AD were included in the randomized clinical trial, who were treated with tonifying kidney decoction (TKD) or AP combined with donepezil hydrochloride (DH) as an intervention measure, while the control group was treated with DH. The total effective rate was the primary outcome, and mini-mental state examination (MMSE) score and activities of daily living (ADCS-ADL) scores were the secondary indicators.

**Results:**

Eventually 30 studies reporting 2236 patients underwent TKD or AP combined with DH were enrolled. In terms of total efficiency, compared with TKD and DH, TKD + DH was significantly preferable. In addition, TKD were classified into 2 categories, namely tonifying kidney with reducing phlegm formulas (TKRP) and tonifying kidney with filling lean marrow (TKFLM). Regarding to MMSE score of TKD, of the 3 interventions, only TKRP + DH (standard mean difference [SMD] = 4.84, 95% confidence interval [CI]: 0.86–8.82) and TKFLM + DH (SMD = 3.93, 95% CI: 1.06–6.80) had significant efficacy over TKFLM (SMD = 4.25, 95%CI: −2.58 to 11.08). Although no difference between TKRP and other groups, its effectiveness was higher than TKFLM + DH and TKFLM (surface under the cumulative ranking curve (SUCRA) = 61.5%). For the ADL score, compared with TKFLM + DH and DH, TKRP + DH had more effective (SUCRA = 70.2%). Regarding to the total effective rates, AP + DH was more statistically better than AP, and AP was statistically better than DH.

**Conclusion:**

TKD or AP in combination with DH are significantly superior in treating AD.

## 1. Introduction

Since the prevalence of various chronic diseases is increasing under the aging global population is aging, Alzheimer’s disease (AD) is one of the chronic degenerative brain disease and the most common etiology of dementia,^[1,2]^ whose main manifestations include amnesia, gradual cognitive impairment, difficulty in fulfilling familiar tasks, impaired understanding, disordered temporal and spatial recognition, with changes in mood and personality. Even in the later stage, there will be loss of mobility, muscle atrophy, difficulty in swallowing and mastication, and systemic complications.^[3,4]^ Therefore, early intervention is an effective measure to defer the pathogenesis and refine the life quality.

Donepezil hydrochloride (DH) is one of the drugs approved by the Food and Drug Administration for the treatment of AD, which has pharmacological effects by affecting cellular and molecular processes of neurodegeneration.^[5]^ DH can selectively inhibit acetylcholinesterase activity, interfere with the expression of AChE-S type, enhance the expression of R-subtype, and produce neuroprotective effect.^[6]^ Although DH is the drug for the treatment of AD, there are still certain problems. For example, it can only delay the progression and lessen the symptoms of AD, but it cannot treat or completely eliminate cognitive dysfunction. In addition, DH has been shown to maintain symptom improvement for only 6 to 12 months, with very limited potential in treating severe and advanced AD to extend the duration of effect. Therefore, it is imperative to find a treatment that can prolong the efficacy and duration of DH action.^[7,8]^

Traditional Chinese medicine (TCM) has long history and plays important roles in the clinical treatment of various diseases. Previous studies have reported that the combination of TCM and DH showed significant differences in activating multiple signaling pathways to promote hippocampal nerve growth compared with DH alone.^[9]^ According to the theory of TCM, kidney essence can supplement the brain marrow and improve the symptoms of forgetfulness and dementia. From the “General Record of Shengji,” tonifying kidney decoction (TKD) was composed of Ginseng, *Astragalus membranaceus*, *Atractylodes macrocephala*, Aconite and other for tonifying kidney and supplementing kidney essence. TKD plays a neuroprotective role by restoring the balance of nerve metabolism and maintaining the expression of synaptic proteins to protect synapses.^[10,11]^ Liu ZH^[12]^ found that the combination of TKD and DH could increase the viability of OLN-93 cells and enhance the activity of PI3K/Akt-MTOR signaling pathway to optimize cognitive function.

Acupuncture (AP), as an important TCM treatment, has been proved to be effective in treating many nervous system diseases including AD. The efficacy of AP has also been recognized by the National Institutes of Health.^[13]^ Wang YY^[14]^ claimed that AP and DH may bring beneficial effects on general cognitive function and activities of daily living (ADCS-ADL) in AD patients than DH (MD = −2.14, 95% CI: −3.69 to −0.59, *P* < .01). Jiang J^[15]^ found in the study of SAMP8 mice that AP and DH could enhance the spatial learning and memory ability of mice, promote the level of glucose metabolism in the brain, and diminish the content of amyloid Aβ in the cortex.

Though previous studies reported the effect and safety of TKD or AP on the treatment of AD, the shortcomings included inadequate detailed AD related outcomes and immaterialized pairwise comparison with DH, which delimiting the implication of clinical practice.^[16]^ Considering the diversity of constitution on different AD patients, the gastrointestinal digestive system of some patients was difficult to bear the cold or heat of TCM, and some patients maybe with needle syncope syndrome. Whether the combined treatment of TCM and western medicine will affect their respective efficacy and increase adverse reactions, the network meta-analysis explored the most effective and safe treatment method where TCM decoction and AP can be used as replacement therapy for clinicians, selecting appropriate TCM methods for patients and combining with western medicine on this basis to achieve twice the result with half the effort.

## 2. Method

### 2.1. Registration

The study protocol was registered with the International Prospective Register of Systematic Reviews (PROSPERO), registration number: CRD42022355910.

### 2.2. Search strategy

This meta-analysis was conducted under the guidance of the PRISMA Guidelines (Preferred reporting items for systematic review and Meta-analysis). By searching the 5 medical databases, including Google Scholars, PubMed, Cochrane Library, Embase, Scopus, Clinical Trials.gov and reference lists of a large number of literatures, as of March 18th, 2022. The language for searching literature was limited to Chinese and English, the mesh terms were as following: (“alzheimer dementia” OR “dementia,” “alzheimer” OR “alzheimer’s disease”) and (“nourishing kidney” OR “the kidney prescriptions” OR “nourishing kidney prescriptions” OR “TCM decoction of nourishing kidney” OR “TCM nourishing kidney decoction”) and (“acupuncture treatment” OR “pharmaco acupuncture treatment” OR “therapy” OR “pharmaco acupuncture treatment”).

### 2.3. Selection process

For eligible studies, 2 investigators (WXC and LK) assessed quality by Review Manager 5.3 by Cochrane bias risk tools. The information collected included author name, age, year of publication, sample size, interventions, total response rate, mean absolute value or mean change in mini-mental State Examination (MMSE) score and ADL score. According to different symptoms in included studies, TKD was divided into tonifying kidney with reducing phlegm formulas (TKRP) and tonifying kidney with filling lean marrow (TKFLM) through TKRP and TKFLM as subgroup analysis to perform the study. Any discrepancy was adjudicated by a senior reviewer (CCL).

### 2.4. Inclusion criteria

The included subjects met the core clinical criteria of AD formulated by the National Institute on Aging and the AD Society. The efficacy of the drug was assessed by the total effective rate, and the degree of cognitive function in AD patients was assessed by the MMSE score. The experimental group used TKD or AP as the intervention measures of the treatment group without other TCM and measures. In the treatment group with TKD, it shall take 300 mL orally every morning and evening after boiling Ginseng, *A membranaceus*, *A macrocephala*, and Aconite. Outcome measures mainly included using MMSE and ADCS-ADL scores to assess cognitive function and behavioral ability in the analysis. The control group be given conventional therapy and donepezi without other TCM and measures.

### 2.5. Exclusion criteria

The exclusion criteria are as follows: Involving other TCM research; Animal studies; Cell research; Repeatedly publication articles, reviews, and conference proceedings.

### 2.6. Data extraction and quality assessment

After searched the full text and contacted experts in the research field to determine any ongoing or missing research, it found unpublished data related to the research to meaningfully combine the estimated results of all studies in the analysis, then collected citations from the literature (forward search) or research from the collected literature (backward search) to obtain relevant research through the Citation Database Elsevier Scopus.

### 2.7. Outcome measures

The relevant results of each study were not extracted in the meta-analysis, and only the crossover results involved in the randomized controlled trials were extracted and analyzed. The main outcome measures were total effective rate, MMSE and ADCS-ADL.

### 2.8. Transitivity and consistency

In the research process, the satisfaction of transitivity hypothesis based on the results of direct and indirect comparison by using logical reasoning, index analysis, data statistics and other methods was objectively tested, it found that the statistical evaluation of direct and indirect comparison results was transitive. The consistency was evaluated by obtaining the confidence intervals (CI) of effect size differences through direct and indirect comparison.

### 2.9. Statistical analysis

For continuous and dichotomous variables, standard mean difference (SMD) with 95% CIs and odds ratios (OR) with 95% CI were generated respectively by network meta-analysis via Stata 16.0. Statistical heterogeneity of fixed effect model was set as *I*^2^ < 50% and *P* > .01. Otherwise, the random effects model would be utilized, then publication bias and small sample effect were assessed by funnel plots. Each outcome was ranked by the surface under the cumulative ranking curve (SUCRA) since the higher the SUCRA could indicate a possibility of superior efficacy. Matrix was formulated to make comparisons among all the interventions to detect whether the difference of SUCRA of each pair reached significance. Inconsistency and consistency were assessed to enhance the stability of the results. *P* < .05 was regarded as statistically significant.

## 3. Results

### 3.1. Literature search findings

**Figure 1.**
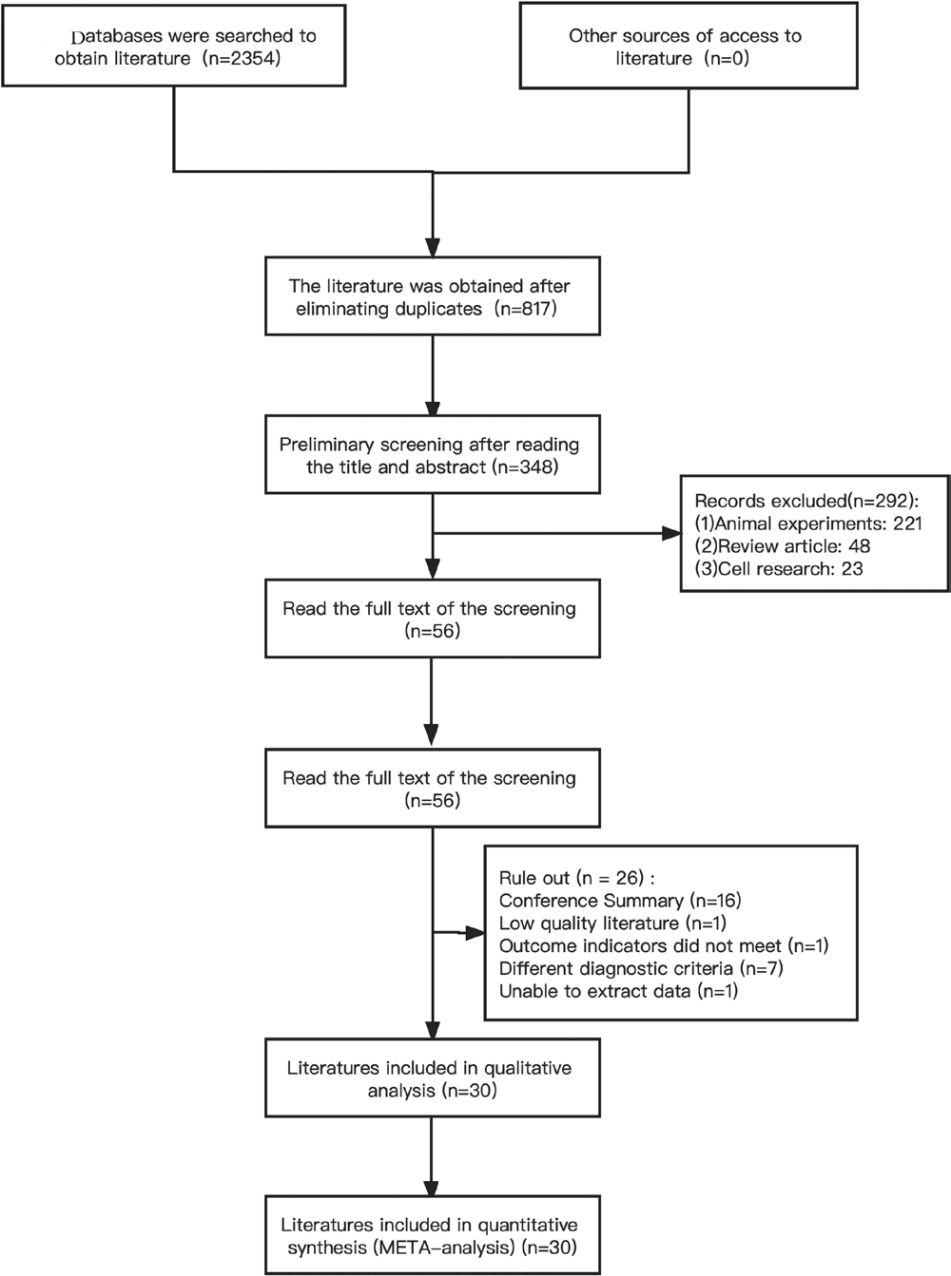
Study selection for a network meta-analysis.

Through an electronic search, 2354 citations were identified, 56 full-text articles were evaluated, 26 studies were excluded, and 30 randomized clinical trials, a total of 2236 patients, were eventually included in the network meta-analysis. The mean age of patients was 55.4 to 72.7 years. All patients were diagnosed with AD (Fig. 1 and Table 1).

**Table 1:** Characteristics of studies included in the meta-analysis.

Included trials	Randomized sample size:I/C	Age (yrs) I/C, mean (SD)	Intervention	Control	Treatment duration (wks)	Jadad scores
Guo et al 2013	62/65	76.9 ± 2.02/75.1 ± 2.98	TKD + DH	DH	12	3
Xue et al 2019	47/47	69.8 ± 5.4/70.4 ± 6.1	TKD + DH	DH	24	3
Zang et al 2012	20/20	69.10 ± 8.05/68.88 ± 8.21	TKD + DH	DH	24	3
Wang et al 2021	40/40	74.78 ± 5.29/75.06 ± 5.15	TKD + DH	DH	12	3
Sang et al 2011	30/30	70. 5 ± 5.6/74	TKD	DH	12	1
Chi et al 2018	47/47	75.36 ± 10.28/74.17 ± 9.31	TKD	DH	24	3
Huan et al 2010	34/34	72.82 ± 7.04/72.29 ± 6.80	TKD + DH	DH	24	1
Huang et al 2018	34/34	72.3 ± 6.8/72.8 ± 7.0	TKD + DH	DH	24	3
Cui et al 2021	34/34	70.23 ± 5.31/71.44 ± 5.82	TKD + DH	DH	24	3
Chen et al 2015	33/33	67.9 ± 13.3/69.2 ± 15.7	TKD + DH	DH	3	1
Li et al 2021	49/49	66.11 ± 9.55/65.79 ± 10.21	TKD + DH	DH	24	1
Yuan et al 2004	20/20	70 ± 4.07/70.9 ± 4.73	TKD + DH	DH	24	1
Pan et al 2017	30/30	66.58 ± 7.48/66.00 ± 6.12	TKD + DH	DH	24	1
Zhang et al 2015	26/25	67.5 ± 8.97/68.72 ± 9.72	TKD + DH	DH	12	3
Zhou et al 2001	34/34	range: 60~87	TKD	DH	12	1
Pan et al 2014	45/46	57.2 ± 9.7/56.9 ± 10.2	TKD + DH	DH	25	4
Liu et al 2013	30/30	74 ± 5/75 ± 6	TKD	DH	12	1
Li et al 2020	30/30	70.42 ± 5.41/72.10 ± 4.16	AP + DH	DH	8	3
Wang et al 2018	55/54	70.88 ± 8.95/70.12 ± 8.99	AP + DH	DH	12	1
Jia et al 2017	100/100	75.11 ± 6.53/74.50 ± 6.83	AP	DH	12	3
Peng et al 2017	25/25	69.4 ± 5.4/69.5 ± 5.3	AP	DH	4	3
Zhao et al 2021	24/24	72.13 ± 3.46/73.33 ± 2.94	AP + DH	DH	8	3
Yang et al 2021	27/27	65 ± 12/65 ± 12	AP + DH	DH	8	1
Ma et al 2021	30/30	63.83 ± 6.24/66.97 ± 7.34	AP	DH	12	3
Su et al 2018	30/30	67.54 ± 4.11/65.87 ± 4.32	AP + DH	DH	12	1
He et al 2018	30/30	67.53 ± 5.54/68.37 ± 5.32	AP + DH	DH	12	3
Liu et al 2008	40/40	69.16 ± 2.12/68.09 ± 6.24	AP	DH	10	3
Lin et al 2016	30/30	69.7 ± 5.36/73.2 ± 4.81	AP + DH	DH	12	4
Li et al 2014	30/30	/	AP + DH	DH	8	1
Li et al 2021	35/35	74.88 ± 4.63/74.17 ± 4.58	AP + DH	DH	24	3

AP = acupuncture, DH = donepezil hydrochloride, TKD = tonifying kidney decoction.

### 3.2. Pair-wise meta-analysis

**Figure 2.**
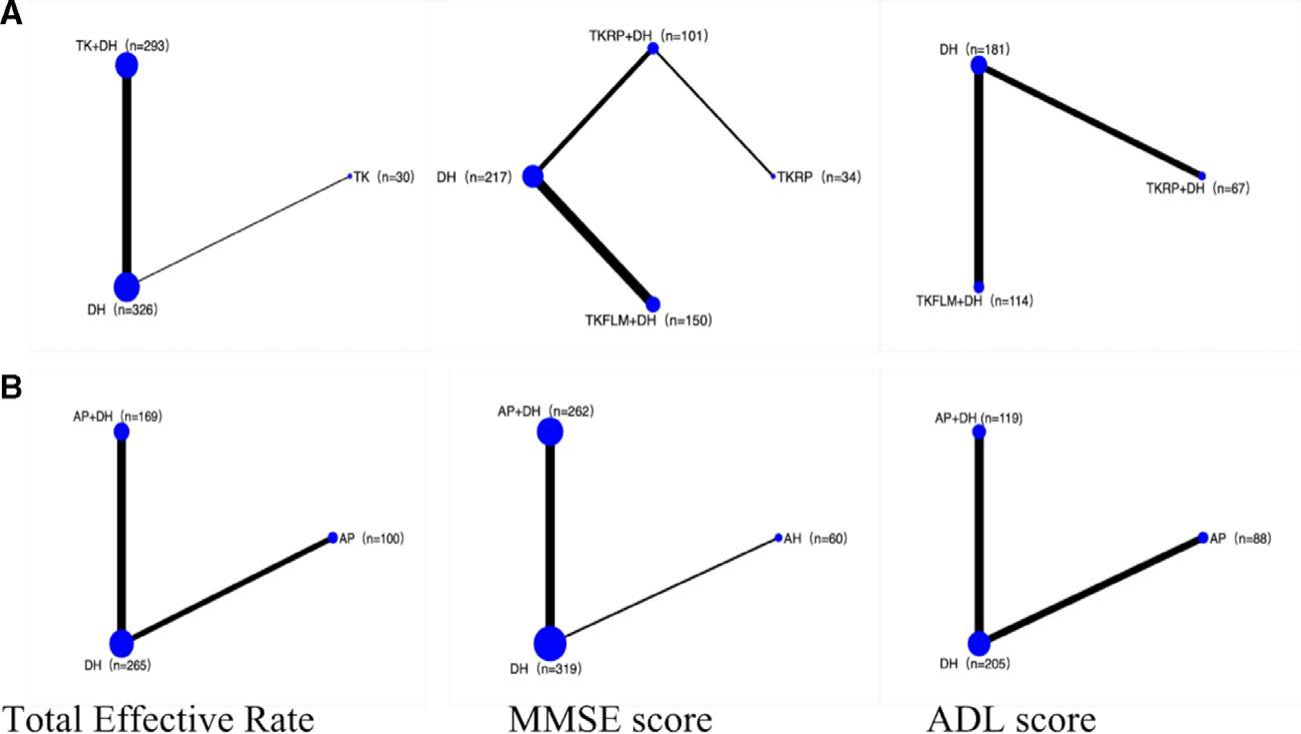
(a) Network of eligible comparisons of TKD and DH, (b) Network of eligible comparisons of AP and DH. The width of the line is proportional to the number of trials compared to each pair of treatments, and the size of each node is proportional to the number of randomly assigned participants (sample size). AP = acupuncture, DH = donepezil hydrochloride, n = sample size, TKD = tonifying kidney decoction, TKFLM = tonifying kidney and filling lean marrow, TKRP = tonifying kidney and reducing phlegm decoction.

Figure 2a showed eligible comparisons of TKD and DH of total effective rates, MMSE score, and ADL score on treating AD. The results showed that the combination of TKD and DH had better effect on improving AD symptoms. Figure 2b showed eligible comparisons of AP and DH of total effective rates, MMSE score, and ADL score on treating AD. The results showed that the combination of AP and DH had better effect on improving AD symptoms.

**Figure 3.**
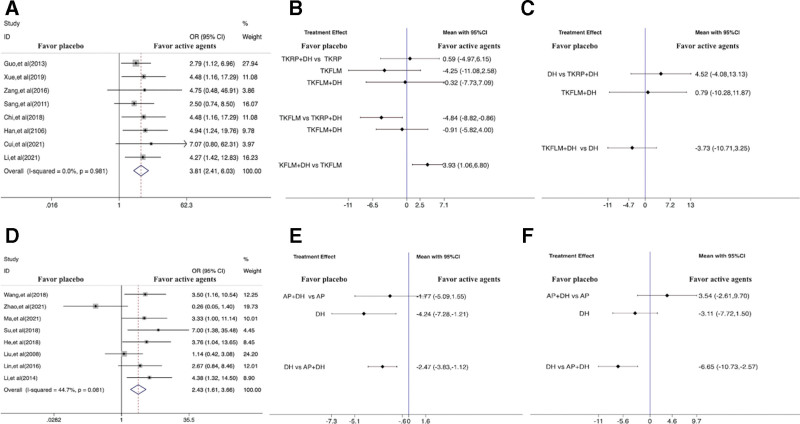
(a) Forest plots of network meta-analysis of TKD on total effective rate, (b) Forest plots of TKD on MMSE score, (c) Forest plots of TKD on ADL score, (d) Forest plots of AP on total effective rate, (e) Forest plots of AP on MMSE score, (f) Forest plots of AP on ADL score. AP = acupuncture, CI = confidence interval, DH = donepezil hydrochloride, OR = odds ratios, TKD = tonifying kidney decoction, TKFLM = tonifying kidney and filling lean marrow, TKRP = tonifying kidney and reducing phlegm decoction.

Figure 3a–c showed forest plots of network meta-analysis of TKD and DH on total effective rate, MMSE score, and ADL score. The results showed that TKFLM + DH was more statistically significant than TKFLM in improving MMSE score (SMD = 3.93, 95% CI: 1.06, 6.80).

Figure 3d–f showed forest plots of network meta-analysis of AP and DH on total effective rate, MMSE score, and ADL score. The results showed that AP + DH was more statistically significant than DH in improving MMSE score and ADL score (SMD = −2.47, 95% CI −3.83, −1.12) (SMD = −6.65, 95% CI: −10.73, −2.57).

### 3.3. Network meta-analysis

Table 2(a) showed the comparison of total effective rates on treating AD. The TKD + DH was the highest (SUCRA = 88%), followed by the TKD (SUCRA = 58.5%), and DH (SUCRA = 3.5%). Compared with TKD and DH, TKD + DH was significantly preferable.

Table 2(b) showed MMSE score in the network comparison of TKD and DH for AD. The TKD were classified into 2 categories, tonifying kidney and reducing phlegm formulas (TKRP) and tonifying kidney and filling lean marrow (TKFLM). TKRP + DH was the highest (SUCRA = 73.7%), followed by TKRP (SUCRA = 61.5%), TKFLM + DH (SUCRA = 60.7%), and TKFLM (SUCRA = 4%). Compared with TKRP, TKRP + DH (SMD = 4.84, 95% CI: 0.86–8.82) was significantly better. Compared TKFLM, TKFLM + DH (SMD = 3.93, 95% CI: 1.06–6.80) was significantly better.

Table 2(c) showed the comparison of ADL score network in treating AD. TKRP + DH was the highest (SUCRA = 70.2%), followed by TKFLM + DH (SUCRA = 64.9%), and DH (SUCRA = 14.8%). Compared with TKFLM + DH and DH, TKRP + DH was significantly better.

Table 3(a) showed the comparison of total effective rates in treating AD. The AP + DH was the highest (SUCRA = 84%), followed by the AP alone (SUCRA = 62.7%), and DH (SUCRA = 3.2%). There were statistically significant differences in all intervention groups. AP + DH was statistically better than AP, and AP was statistically better than DH.

Table 3(b) showed the comparison of MMSE score in treating AD. The AP was the highest (SUCRA = 92.4%), followed by the AP + DH (SUCRA = 57.5%), and DH (SUCRA = 0.2%). Compared with DH, AP (SMD = 4.24, 95% CI: 1.21–7.28) was significantly better. Compared with DH, AP + DH (SMD = 2.47, 95% CI: 1.12–3.83) was significantly better.

Table 3(c) showed the comparison of ADL score in treating AD. Compared with DH, the AP + DH (SMD = 6.65, 95% CI: 2.57–10.73) was significantly better.

General and loops inconsistency was not applicable in the above outcomes since there were no closed loops.

In term of TKD treatment of AD, funnel plot showed the small sample study effect existed in comparison of TKRP + DH versus TKFLM in term of MMSE score. Comparison of TKRP + DH versus DH in term of ADL score has the small sample study effect exists. Funnel plot showed that the small sample study effect existed in comparison of AP + DH versus DH in term of MMSE and ADL score in AP treatment of AD.

## 4. Discussion

As the first network meta-analysis comparing the effects of TKD or AP combined with DH, the results showed that TKD combined with DH could significantly ameliorate clinical symptoms of AD, including decreased memory and body dysfunction. AP combined with DH were suited effectively in deferring the accumulation of Aβ or tau protein and intellectual injure.

Although AD as a neuro-degenerative disease, many studies have found that the pathogenesis of AD was closely related to the kidney. Ghiso^[17]^ has confirmed previously that the kidney was involved in Aβ clearance, and the serum Aβ concentration in patients with chronic kidney disease was significantly increased. One study had shown that there was a 2-way communication between kidney and brain.^[18]^ There are many factors affecting kidney brain communication system, including oxidative stress of cells when decreased renin-angiotensin system function. In addition, the central nervous system conduction disorder will not only lead to traumatic brain injury and migraine, but also affect the inflammatory reaction of renal vascular endothelial cells.

In the study, TKD was divided into 2 subgroups, TKRP and TKFLM. TCM theory regarded the human body as a unified whole, so other symptoms of patients should be considered when treating AD. Therefore, on the basis of TKD, it added some TCM according to different accompanying symptoms of patients. TKRP mainly added Rhizoma Arisaematis, Pinellia ternata, Semen Brassicae Albae and Aster Tataricusand. TKFLM mainly added Semen Cuscutae, Epimedium Brevicornu Maxim, Polygonum Multiflorum and Lycium chineses. In this study, although the TKRP and TKFLM can improve MMSE and ADL score, the combination of TKRP and DH was significantly better than TKFLM and DH (SUCRA = 73.7%, SUCRA = 70.2%).

DH, as a second-generation cholinesterase inhibitor used for mild to moderate AD, could reversely inhibit acetylcholinesterase to facilitate cholinergic nerve transmission.^[19]^ In previous AD meta-analysis studies, TKD + DH enhanced overall effective rate pass beyond DH alone (OR 2.74, 95% CI: 1.55–4.85, *P* = .0006).^[16]^ When combined with other TCM decoction, the cognitive decrement of moderate and severe patients were significantly retarded, which was generally stable within 2 years in mild patients.^[20]^

In previous studies, it was found that the combination of TKD and DH would increase the incidence of adverse reactions in patients, which was considered to be related to the gastrointestinal intolerance to drugs.^[21]^ One study found that the combination of TKD and DH could increase ADL score, but the sound mechanism had not been established and remains to be studied.^[22]^

AP, a 3000-year treatment, has shown positive results for several neurological disorders, including AD. Studies reported that AP could up-regulate synaptophysin and postsynaptic dense-95 protein, significantly promoted working memory and synaptic plasticity, dwindled neuro-inflammation and synaptic ultrastructural degradation in 5XFAD mice.^[23,24]^ Jing J^[15]^ demonstrated that AP combined with DH could ameliorate spatial learning and memory ability, brain glucose metabolism level and cortical amyloid Aβ content. One study showed that^[25]^ AP stimulation can improve learning and memory ability by maintaining neuronal mitochondrial integration, promoting the normal function of APP, reducing Aβ plaque and increasing the transmission of acetylcholine in the hippocampus of APP mutant mice.

It summarized the characteristics of included literature (Supplemental Digital Content [Table S1, http://links.lww.com/MD/H685]), such as MMSE score, duration of illness and advantage event. It found that TKD or AP, whether used alone or in combination with DH, can assuage symptoms and curative time for AD patients with different degrees of symptoms. But for patients with moderate or severe AD, TKD combined with DH is precisely suitable treatment for AD. For patients with mild or moderate AD, AP combined with DH is the suitable treatment for AD, but whether TKD + AP + DH can be used and whether it can achieve superior therapeutic effect needs to be studied on a larger sample size and scale.

In terms of adverse reactions mentioned in the included studies, TKD has no adverse reactions except for people with gastrointestinal diseases, since it will lead to nausea and stomach pain. AP has no adverse reactions to patients without a history of needle syncope.

## 5. Limitation

As for the limitations, firstly, the number of studies and the included patients were relatively small. When analyzing the literature in this network meta-analysis, it extracted the mean, SD, baseline and size values of the samples used for analysis and last observation. However, some studies have lost their data, making the number of existing studies even smaller. Secondly, some of the literature did not mention the allocation method, resulting in the low quality of the research literature. Specific interventions and patient populations vary from study, which can lead to heterogeneity. Finally, it did not extract adverse reactions, because only 3 literatures mentioned the occurrence of adverse reactions.

## 6. Conclusion

In this network meta-analysis, it found that TKD or AP in combination with DH were significantly efficient than TKD or AP in the treatment of AD.

**Table 2(a):** Matrix of pairwise comparison among TKD on total effective rate (shown as OR and 95% CI).

	TKD + DH	TKD	DH
SUCRA (%)	88	58.5	3.5
TKD + DH	0	0.62 (0.17, 2.34)	0.25 (0.15, 0.41)
TKD	1.60 (0.43, 6.01)	0	0.40 (0.12, 1.36)
DH	4.01 (2.44, 6.60)	2.50 (0.74, 8.50)	0

CI = confidence interval, DH = donepezil hydrochloride, OR = odd ratio, SUCRA = surface under the cumulative ranking curve, TKD = tonifying kidney decoction.

**Table 2(b):** Matrix of pairwise comparison among TKD on MMSE score (shown as SMD and 95% CI).

	TKRP + DH	TKRP	TKFLM + DH	TKFLM
SUCRA (%)	73.7	61.5	60.7	4
TKRP + DH	0	−0.59 (−6.15, 4.97)	−0.91 (−5.82, 4.00)	−4.84 (−8.82, −0.86)
TKRP	0.59 (−4.97, 6.15)	0	−0.32 (−7.73, 7.09)	−4.25 (−11.08, 2.58)
TKFLM + DH	0.91 (−4.00, 5.82)	0.32 (−7.09, 7.73)	0	−3.93 (−6.80, −1.06)
TKFLM	4.84 (0.86, 8.82)	4.25 (−2.58, 11.08)	3.93 (1.06, 6.80)	0

CI = confidence interval, DH = donepezil hydrochloride, OR = odd ratio, MMSE = mini-mental state examination, SMD = standard mean difference, SUCRA = surface under the cumulative ranking curve, TKD = tonifying kidney decoction, TKFLM = tonifying kidney with filling lean marrow, TKRP = tonifying kidney with reducing phlegm formulas.

**Table 2(c):** Matrix of pairwise comparison among tonic kidney decoction on ADL score (shown as SMD and 95% CI).

	TKRP + DH	TKFLM + DH	DH
SUCRA (%)	70.2	64.9	14.8
TKRP + DH	0	0.79 (−10.28, 11.87)	4.52 (−4.08, 13.13)
TKFLM + DH	−0.79 (−11.87, 10.28)	0	3.73 (−3.25, 10.71)
DH	−4.52 (−13.13, 4.08)	−3.73 (−10.71, 3.25)	0

ADL = activity of daily living scale, CI = confidence intervals, DH = donepezil hydrochloride, MMSE = mini-mental state examination, OR = odds ratios, SMD = standard mean difference, SUCRA = the surface under the cumulative ranking curve, TKD = tonifying kidney decoction, TKFLM = tonifying kidney and filling lean marrow, TKRP = tonifying kidney and reducing phlegm decoction.

**Table 3(a):** Matrix of pairwise comparison among AP on total effective rate (shown as OR and 95% CI).

AP + DH	AP + DH	AP	DH
SUCRA (%)	84	62.7	3.2
AP + DH	0	0.74 (0.22, 2.51)	0.35 (0.16, 0.79)
AP	1.36 (0.40, 4.62)	0	0.48 (0.19, 1.20)
DH	2.83 (1.27, 6.29)	2.08 (0.83, 5.23)	0

AP = acupuncture, CI = confidence intervals, DH = donepezil hydrochloride, OR = odds ratios, SUCRA = the surface under the cumulative ranking curve.

**Table 3(b):** Matrix of pairwise comparison among AP on MMSE score (shown as SMD and 95% CI).

	AP	AP + DH	DH
SUCRA (%)	92.4	57.5	0.2
AP	0	−1.77 (−5.09, 1.55)	−4.24 (−7.28, −1.21)
AP + DH	1.77 (−1.55, 5.09)	0	−2.47 (−3.83, −1.12)
DH	4.24 (1.21, 7.28)	2.47 (1.12, 3.83)	0

AP = acupuncture, CI = confidence intervals, DH = donepezil hydrochloride, MMSE = mini-mental state examination, OR = odds ratios, SMD = standard mean difference, SUCRA = the surface under the cumulative ranking curve.

**Table 3(c):** Matrix of pairwise comparison among AP on ADL score (shown as SMD and 95% CI).

	DH	AP	AP + DH
SUCRA (%)	95.3	48.4	6.2
DH	0	3.11 (−1.50, 7.72)	6.65 (2.57, 10.73)
AP	−3.11 (−7.72, 1.50)	0	3.54 (−2.61, 9.70)
AP + DH	−6.65 (−10.73, −2.57)	−3.54 (−9.70, 2.61)	0

ADL = activity of daily living scale, AP = acupuncture, CI = confidence intervals, DH = donepezil hydrochloride, SMD = standard mean difference, SUCRA = the surface under the cumulative ranking curve.

## Acknowledgments

This study was supported by the School of Food and Pharmaceutical Engineering, Zhaoqing University and department of Neurology, Guangdong Hospital of Traditional Chinese Medicine

## Author contributions

X.W. finished the data analysis. C.C., K.L. and X.C. wrote and revised the manuscript. X.W. and X.J. conceived and designed the study. S.Q. acquired funding supports and approved the final manuscript as submitted.

**Data curation:** Kuan Lu, Xiao-Qian Jin.

**Investigation:** Xin-Chen Wang, Chen-Liang Chu, Kuan Lu, Xi Chen, Xiao-Qian Jin.

**Software:** Xin-Chen Wang, Kuan Lu, Xi Chen, Xiao-Qian Jin.

**Supervision:** Shi-Jian Quan.

**Visualization:** Chen-Liang Chu, Xiao-Qian Jin, Shi-Jian Quan.

**Writing – original draft:** Xin-Chen Wang.

**Writing – review & editing:** Chen-Liang Chu, Xi Chen, Shi-Jian Quan.

## Supplementary Material
