# Supplementary material for: The role of tonifying kidney decoction and acupuncture in the treatment of Alzheimer’s disease: A network meta-analysis
Source: Medicine (Baltimore). 2022 Nov 18;101(46):e31243. doi: 10.1097/MD.0000000000031243 (PMC9678600; doi:10.1097/MD.0000000000031243)
Supplement: Supplementary file 1 [file medi-101-e31243-s001.pdf]

Supplementary Table

Characteristics of included literature

Author, year	Patients (no. I/C)	MMSE score(I/C)	Duration of illness（Year，I/C）	Medication Intervention (I/C)	Adverse event (no.)
Guo 2013[1]	62/65	5-12	3-6	TKD+DH/DH	NA
Xue 2019[2]	47/47	11-25/12-26	6.4±1.7/6.9±1.3	TKD+DH/DH	NA
Zang 2016[3]	20/20	NA	38.28±9.19/39.01 ± 9.25	TKD+DH/DH	NA
Wang 2021[4]	40/40	NA	2.05±0.76/2.10±0.69	TKD+DH/DH	NA
Sang 2011[5]	30/30	NA	0.5-5	TKD/DH	NA
Chi 2018[6]	47/47	NA	4.93±1.02/5.27± 1.15	TKD+DH/DH	NA
Han 2016[7]	34/34	NA	NA	TKD+DH/DH	NA
Huang 2018 [8]	34/34	16-22	NA	TKD+DH/DH	1
Cui 2021[9]	34/34	11-24/12-26	5.93 ± 1.37/6.01 ± 1.42	TKD+DH/DH	NA
Chen 2015[10]	33/33	16.8±4.3/15.2±4.7	3.9±2.9/3.7±3.1	TKD+DH/DH	NA
Li 2021[11]	49/49	11.8±3.4/12±4.69	4.38±0.42/4.59±0.54	TKD+DH/DH	NA
Yuan 2004[12]	20/20	15.9±4.1/17.1±4.1	6.5 ±1.9/6.6±2.4	TKD+DH/DH	NA
Pan 2017 [13]	26/27	22.54±2.25/22.59±2.37	0-3	TKD+DH/DH	NA
Zhang 2015[14]	26/25	14.38±4.92/13.92±4.89	0.5-3	TKD+DH/DH	NA
Zhou 2011[15]	34/34	12.06±5.31/12.91±5.71	NA	TKD/DH	NA
Pan 2014[16]	49/49	10-24	5.7±4.9/5.9±5.2	TKD/DH	NA
Liu 2013[17]	30/30	14.2±1.8/15.2±2.5	NA	TKD/DH	NA
Li 2020[18]	30/30	16.89±2.59/16.69±2.42	17.57±/5.06/16.62±6.53	AP+DH/DH	NA
Wang 2018[19]	55/54	19.58±2.8/19.44±2.89	2.16±0.26/2.05± 0.25	AP+DH/DH	NA
Jia 2017[20]	43/44	NA	4.46±0.99/4.42±1.06	AP/DH	NA
Jing 2017[21]	25/25	15.53±6.17/16.71±2.63	7.5±1.8/7.6±1.7	AP+DH/DH	NA
Zhao 2021[22]	24/24	19.43±1.53/19.21±1.5	23.79±1.69/24.1±2.88	AP+DH/DH	1
Yang 2021[23]	27/27	15.05±2.69/14.57±2.36	2.4±0.7/2.3±0.7	AP+DH/DH	NA
Ma 2021[24]	30/30	19.23±2.37/19.23±2.34	1.43±0.37/1.58±0.42	AP/DH	NA
Su 2018[25]	30/30	14.03±3.18/15.4±2.59	4.67±1.99/4.97±2.16	AP+DH/DH	NA
He 2018[26]	30/30	16.27±3.93/15.87±4.18	1.82±0.74/1.94±0.64	AP+DH/DH	NA
Liu 2016[27]	40/40	18.92±4.08/19.02±3.86	0.82±0.43/0.84±0.22	AP/DH	NA
Lin 2016[28]	30/30	24.5±4.2/22.7±3.6	NA	AP+DH/DH	NA
Li 2014[29]	30/30	16.27±0.71/16.67±0.73	NA	AP+DH/DH	NA
Li 2021[30]	35/35	18.51±1.14/ 18.34±1.13	4.17±1.04/4.21±1.07	AP+DH/DH	NA

TKD:Tonifying kidney decoction
AP:Acupuncture
NA:Not Applicable
